# Retraction: Upregulation of C terminus of Hsc70-interacting protein attenuates apoptosis and procoagulant activity and facilitates brain repair after traumatic brain injury

**DOI:** 10.3389/fnins.2023.1330870

**Published:** 2023-11-02

**Authors:** Frontiers Editorial Office

Following publication, concerns were raised regarding the integrity of the images in the published figures. Specifically, image duplication concerns were identified in Figure 7E. The authors failed to provide a satisfactory explanation during the investigation, which was conducted in accordance with Frontiers' policies. As a result, the data and conclusions of the article have been deemed unreliable and the article has been retracted. The authors agree to this retraction.

